# When thinking you are better leads to feeling worse: Self-other asymmetries in pro-social behavior and increased anxiety during Covid-19

**DOI:** 10.1371/journal.pone.0291329

**Published:** 2023-09-14

**Authors:** Chelsea Helion, Virginia Ulichney, David V. Smith, Johanna Jarcho

**Affiliations:** Department of Psychology and Neuroscience, Temple University, Philadelphia, PA, United States of America; Macedonian Academy of Sciences and Arts, THE FORMER YUGOSLAV REPUBLIC OF MACEDONIA

## Abstract

Self-serving biases (e.g., beliefs that one tends to perform better than peers) are generally associated with positive psychological outcomes like increased self-esteem and resilience. However, this tendency may be problematic in the context of collective action problems, wherein individuals are reliant on others’ pro-social behaviors to achieve larger goals. We examined this question in the context of the Covid-19 pandemic, and recruited participants for an online study (*n* = 1023) from a university community in Spring 2020. We found evidence for self-peer asymmetries in Covid-related knowledge and restriction behavior, such that participants reported that they knew more about Covid-related symptoms, were doing more to stop the spread of the disease, and were more pro-socially motivated in doing so than peers. Actual peer reports indicated that these were overestimations. This self-enhancement comes with a cost: the perceived self-peer restriction behavior asymmetry had an indirect effect on the positive relationships both from Covid-specific worry and from perceived stress to general anxiety symptom intensity during the early lockdown period. People tended to have more severe symptoms of anxiety when they were more worried about Covid-19 and when they reported greater perceived stress, especially when they underestimated others’ contributions to public health action relative to their own. This suggests that lack of trust in others’ pro-sociality may be personally maladaptive for mental health.

## Introduction

Whether it’s thinking we are better drivers, better partners, or simply better people, we tend to evaluate ourselves more favorably than we do others [1–3]. This bias potentially creates a psychological tension in densely networked societies, wherein we often *have* to rely on others to progress in any domain [4]. What’s more, people tend to self-enhance on the values that they prioritize, and these personally-held values can in turn be influenced by broader societal principles. For example, Sedikides, Gaertner, and Toguchi (2003) found that, when engaging in social comparison, those in the United States (where independence tends to be more societally prioritized) *and* anyone who prioritized independence often played up their own individualist traits relative to others [5]. In contrast, people in Japan (where interdependence tends to be more societally prioritized) *and* individuals who valued interdependence alike tended to inflate their personal collective-focused attributes compared to others [5, 6]. Especially in environments wherein individualism is societally prioritized (e.g., the U.S.), if people are surrounded by those they tend to perceive as less competent, less knowledgeable, and more selfish than themselves [7], they may also be pessimistic about the likelihood of collective action to bring about positive change. For example, in the United States uniquely, people tend to find their own violations of protective Covid-19 protocols (e.g., social distancing, mask-wearing) more acceptable than others’, a perception which is not shared, for example, by their counterparts in China [8].

The Covid-19 epidemic strongly reflects this tension between individual goals and collective action—seldom have we been so reliant on others’ behaviors to quickly produce positive societal outcomes. As such, those who fail to act on behalf of collective protection (e.g., failure to vaccinate, provided adequate access to do so) consequently tend to incur reduced generosity from those who choose to act in the interest of the collective (e.g., by vaccinating; [9]). Although action to mitigate the impact of a societal challenge may be enhanced by some social motivations (e.g., norms, moralization), it also may be diminished through social comparison as self-serving biases cause one to inaccurately believe they are doing more to mitigate the issue than peers [10]. While self-serving biases can be adaptive, they may be psychologically suboptimal in situations that require relying on others for positive outcomes (i.e., collective action problems). We test this hypothesis by examining whether, in the setting of a U.S. university community, the individuals who are most likely to think they are doing more than their peers are also the ones that psychologically fare worse when they are in a situation (i.e., the Covid-19 pandemic) wherein achieving a positive outcome requires reliance on the behaviors of others.

Self-serving bias is associated with higher self-esteem [11], underlies predictions for rosier futures [12], and dulls recollections of less-than-ideal pasts [13]. However, this tendency can also lead to undue cynicism [14] and result in inaccurate perceptions of both oneself and others [15]. For example, individuals claim more responsibility for positive joint outcomes than is logically possible [2, 14]. This kind of self-serving bias is generally benign—absent the occasional argument with a partner that might come from overclaiming one’s contribution to the housework, these biases may typically boost positivity and increase productivity [16]. However, they may become problematic when positive societal outcomes depend on the behaviors of others. Data examined in the present study come from the first wave (collected in March/April 2020) of a larger longitudinal project examining multifaceted impacts of the Covid-19 pandemic within a United States university community. Past research examining data collected at a later wave of the same longitudinal project (in October 2020) found that when individuals felt they were more concerned about social issues than peers—Covid-19, climate change, and racial injustice alike—they also felt that they took more action, which may have implications for approaches in mitigating social issues more broadly [17]. In this article, we examine whether self-serving biases arise when it comes to Covid-19 restriction behaviors and knowledge, and if so, whether these have negative implications for mental health. If individuals are overly confident in their abilities to predict others’ behavior [18, 19], but these predictions are overly cynical [14], then outcomes that rely on others doing the “right thing” may seem unlikely to occur. Individuals who think more of themselves and less of their peers may thus be particularly pessimistic about the likelihood of resolving collective action issues, and therefore more prone to higher levels of anxiety.

The Covid-19 pandemic presents an important example of a collective action problem. To successfully contain the virus, individuals had to collectively practice behaviors perhaps novel to them early in the pandemic, like mask-wearing and social-distancing [20]. Some have posited that one of the most powerful predictors of collective action success in Covid-19 is trust [21]—both horizontal trust in each other [22] and vertical trust in our institutions [23]. The former—the trust that we place in others to do the right thing for the benefit of the group—is the focus of the research presented below. We expect that individuals will report increased engagement in behaviors that reduce the spread of Covid-19 relative to their peers. Moreover, we predict that this lack of trust in peers will be related to increased concern (e.g., general stress, Covid-specific worry), and be associated with heightened anxiety during the pandemic. Some prior research suggests a potentially dangerous “optimism bias” in the context of the pandemic, wherein people may underestimate the riskiness of their own actions for spreading Covid-19 relative to others’ [21]. Here, we directly test whether people perceive they are doing more than others to mitigate Covid-19 and thus demonstrate relatively low levels of horizontal trust. We furthermore examine impacts of these social perceptions on personal mental health.

It is important to note that in this article, we take a two-sided approach to examine experiences of stress during the pandemic: 1) generalized, perceived stress and 2) Covid-specific worry. Both Covid-specific worry and perceived stress are conceptualized as experiences that arise relatively temporarily. Although they could have implications for mental health (including anxiety levels, as we test here), they are not always maladaptive on their own [24]. We examine both generalized perceived stress independent of Covid-specific stressors and Covid-specific worry unique to virus-related stressors. Although stress can be considered a physiological state [25], we focus here on more subjective stress processes reported through the validated Perceived Stress Scale [26]. Throughout the manuscript, we will refer to this generalized stress measure simply as “perceived stress”, and will refer to aggregate behavioral and subjective experiences of distress related to Covid-19 as “Covid-specific worry”. In contrast, anxiety is considered a more chronic, enduring experience characterized by excessive levels of negative affective experiences that can result in a clinical diagnosis at its most severe and maladaptive expression [27, 28]. As a result, we measure anxiety levels using cross-cutting symptom criteria laid out in the DSM-5 [29] (see *Measures* for details).

In this article, we test a set of pre-registered hypotheses and exploratory hypotheses related to them. Exploratory and pre-registered hypotheses are denoted as such following our pre-registration (https://aspredicted.org/blind.php?x=HGQ_8XB). We examine three areas of predicted self-other asymmetry with regard to the Covid-19 pandemic: 1) that individuals will report being more knowledgeable about Covid-19 than their peers (Hypothesis 1A, exploratory); 2) that individuals will report having made more Covid-19 behavioral changes relative to their peers (Hypothesis 1B, exploratory); and 3) that individuals will report they will be less likely to become infected with Covid-19 than their peers (Hypothesis 1C, exploratory). Turning first to Covid-related knowledge, individuals tend to overestimate their knowledge in domains where absolute knowledge is high relative to domains where it is low [30]. As the pandemic had received a great deal of coverage in the media at the time of data collection [31], we predicted that Covid-19 may be a domain where absolute knowledge is relatively high, and that individuals would subsequently overestimate their knowledge relative to peers.

We further predicted that individuals who showed a more pronounced asymmetry (as indexed by reporting greater Covid-19 related knowledge and protective behavior than peers) would report higher levels of anxiety and perceived stress compared to those with smaller self-other asymmetries (Hypothesis 1D, pre-registered H1). Anxiety symptoms have been highly prevalent during the Covid-19 pandemic [32–35]. There are many possible reasons for this increase, including increased media and news consumption [36], economic concerns [37], and concern about individual/familial health [38]. We posit that another contributor may be the lack of influence individuals have over the behaviors of others paired with the centrality of others’ behaviors in determining Covid-19 related outcomes. We expected that individuals who showed a larger self-other asymmetry in Covid-related knowledge and behavior would report higher levels of Covid-specific worry, general anxiety symptoms, and perceived stress during the initial lockdown period.

What’s more, related to the prosocial implications of taking public health precautions outlined prior, Covid-19 related behaviors are morally-relevant. At the time of data collection, individuals who were more pro-socially inclined reported wearing a mask more frequently and perceived others who wore masks as being more pro-social than those who did not [39]. This suggests that Covid-19-related behavior is moralized, and would have meaningful implications for self-other asymmetry predictions, as individuals tend to make highly self-serving asymmetric predictions about actions that have moral implications [3]. We predicted that individuals would report restricting their behavior to a greater extent than peers (as noted in H1B), and moreover, would be more likely to endorse doing so for pro-social as compared to self-focused reasons (Hypothesis 2, exploratory). Our pre-registered secondary hypothesis was outside scope of present research so will not be discussed further. Our exploratory H2 instead examines more relevant topics to H1 and H3 (on perceived self-other asymmetries in prosocial motivation for taking Covid-related action) that better match the focus of the article.

Lastly, we predicted that individuals would be overly pessimistic about their peers’ knowledge and behavior such that the predicted difference between self and peer Covid-19 related knowledge and behavioral restrictions (e.g., social distancing actions) would be larger than the actual differences observed in the sample (respectively Hypotheses 3A & 3B, pre-registered H3).

Notably, prior research has found a *negative* relationship between the tendency to self-enhance relative to one’s peers and anxiety [40]. However, we predicted the opposite effect in this context, given that surviving a global pandemic necessitates reliance on others. If individuals believe that their peers are doing less than they are to contain the spread of Covid-19, then they may also feel more worried about Covid-19 and generally stressed, and thus more anxious during the lockdown period. To examine this question, we tested two exploratory indirect effects models wherein the magnitude of self-peer asymmetry in Covid-19-related restrictive behaviors mediated the relationship between 1) Covid-specific worry and 2) perceived stress with general anxiety reported during the early stages of the Covid-19 pandemic (Hypothesis 4, exploratory).

## Materials and method

The data presented here is part of the first wave of a longitudinal study conducted at a large northeastern university in the U.S. Six waves of data were ultimately collected for the broader project from which this data originates between March and October of 2020. To test the hypotheses laid out in this article, we analyze only the first wave of data collection. This is because the first wave was collected very early on in the pandemic, which minimized potential confounds relevant to our research questions (e.g., changes in Covid-relevant information related to things like transmission vectors, best public health practices, or vaccine development/availability). At that time, participants likely had approximately similar levels of Covid-19 information and exposure and there were fairly circumscribed Covid-19 mitigation behaviors [41]. The study was approved by the Temple University Institutional Review Board (protocol number 26855) and was administered via REDCap, a secure web application for building and managing online surveys and databases. Consistent with IRB approved study procedures, prior to data acquisition, all participants provided electronic informed consent. All participants were members of the larger university community (e.g., undergraduates, graduate students, faculty, etc.). This is perhaps an ideal sample in which to study the nature and accuracy of self-peer asymmetries as we can compare individual estimates of the average population with what the average population is actually reporting. For example, we can compare what an individual undergraduate believes their peers are doing with what undergraduate students are actually reporting in the sample. This provides an ecologically-valid, concrete point of reference from which to examine whether individuals’ beliefs about where they stand relative to their peers are accurate. Undergraduate participants were compensated through course credit based on department-level acceptance of credits. All participants were entered into a raffle at each wave of the study. Participants were recruited via university listservs, and their participation entered them in a lottery to win a $100 Amazon gift card.

The first wave of data collection was completed on 3/25/20-4/4/20, approximately 2–3 weeks following the closure of the university on 03/11/2020, and consisted of 1852 participants (1170 female, *M*_*age*_ = 24.03, *SD*_*age*_ *=* 9.24, age range = 18–76). Given the long-term longitudinal aims, we recruited as many participants as possible for the first wave. The total first-wave sample consisted of 1386 undergraduate students (74.84%), 166 graduate students (68 Masters students, 98 PhD students; 8.96%), 142 staff members (7.67%), 80 faculty members (43 Non-Tenure Track, 16 Tenure-Track; 4.32%), 26 individuals who identified as “Other” (1.4%), and 52 (2.81%) who did not indicate the nature of their affiliation with the university. We only included participants who filled out (rather than leaving unanswered or selecting “prefer not to respond”) all of the measures of interest (Covid-related behavioral restriction and risk-assessment), leaving us with a final sample of 1023 participants (697 female, *M*_*age*_ = 23.61, *SD*_*age*_ = 8.52, age range = 18–71). We believe that this sample size is appropriate given that all analyses were conducted within-participant, providing sufficient power for even more complex analyses [42].

### Measures

We collected a number of measures within this sample, but for the purposes of this manuscript, only focused on the variables listed here. Measures, and items within them, were both presented to participants in a consistent order, with Covid-specific measures first followed by other measures relevant to health, well-being, and social relationships. The entire study took about one hour to complete. When asking participants to recall behaviors, we asked specifically about the past two weeks as that approximately matched up to the initiation of Covid-19 protocols at the university level and ensured consistency across both participants’ considerations and measures (e.g., validated clinical anxiety symptom and perceived stress scales also asked about the past two weeks). Hypotheses and analyses labeled as “pre-registered” (i.e., not exploratory) were preregistered on AsPredicted.org: https://aspredicted.org/blind.php?x=/HGQ_8XB. We note that as of the date of pre-registration submission, no analyses had been completed, although formal data collection had finished and data had been accessed for the data examined in the present article (collected from 03/25/2020–04/04/2020). The rapid sequence of lock-downs early in the pandemic made data collection time-sensitive. Our intention in pre-registering these analyses prior to examining data, but after data collection was to reduce researcher degrees of freedom in the analytic process.

### Covid-19 knowledge

Accuracy of Covid-19 symptoms and general knowledge were evaluated based on the information that was known at the time—March/April 2020. We acknowledge that knowledge about common symptomology for Covid-19 has changed and is still developing as medical researchers learn more about the disease [41].

*Perceived self-peer Covid-19 knowledge asymmetry*. To assess perceived relative knowledge regarding Covid-19, participants were asked to indicate “how knowledgeable they were about Covid-19 compared to their peers” on a scale from 0 = “Know much less” to 6 = “Know much more”. On this scale, the midpoint (3) would indicate an assessment of equivalent knowledge relative to peers. This measure asked directly whether participants felt that they knew more about Covid-19 than peers. As such, we could uniquely use this measure as an indicator of perceived self-peer Covid-19 knowledge asymmetry rather than calculating a difference score between separately-reported self and peer estimates as we do for other measures (e.g., Covid self-peer risk assessment).

*Actual Covid-19 symptom knowledge*. To assess actual knowledge relative to one’s peers, we asked participants to indicate the three most common symptoms of Covid-19 from the following list (the correct answers–symptoms most commonly associated with the wave of Covid-19 infecting the population in March/April 2020—have been bolded): fatigue, nausea, **shortness of breath**, sneezing, diarrhea, constipation, **cough**, runny nose, **fever**, body aches, and ulcers in the mouth; [41]). For each participant, we summed the number of symptoms that they correctly identified and the number of symptoms that they incorrectly identified, and created a difference score by subtracting the incorrect number from the correct number, such that higher numbers indicate greater knowledge of Covid-19 symptomatology.

*Covid-19 general knowledge*. To further assess Covid-related knowledge at a time in which it was still novel and wherein it could be challenging for the public to delineate between accurate and inaccurate information [21], participants were asked to read each of the following statements, and to indicate the extent to which they believed these statements about Covid-19 (correct statements as identified at the time of data collection have been bolded): 1) **“Someone can transmit it, even if they do not show symptoms”**, 2) “Your dog or cat can transmit it to you”, 3) **“Standard surgical masks prevent you from becoming infected”**, 4) “It mutated from the common cold”, 5) “It was probably made in the lab”, 6) “It is less deadly than the annual flu”, 7) “You can become infected from eating at a Chinese restaurant”, 8) “Vitamin C supplements will help keep you from being infected”. All statements were evaluated on a scale from 0 = “Proven False” to 6 = “Proven True”. For each participant, we calculated their average response for the correct items and their average response for the incorrect items, and created a difference score by subtracting their incorrect average from their correct average, such that higher numbers indicate greater knowledge of Covid-19.

*Assessing self-peer knowledge asymmetries*. To examine whether an assessment of being more or less knowledgeable about Covid-19 reflected reality, we calculated the average Covid-19 symptom knowledge and average Covid-19 transmission knowledge for each peer group in the sample (i.e., undergraduates, graduate students, faculty, etc.). We then compared the individual participant’s averages to their peer group averages. To do so, we subtracted the peer average from each participant’s knowledge score, such that higher numbers indicate *actually* being more knowledgeable about Covid-19 relative to one’s peers. The difference score for symptom-knowledge was moderately skewed (skewness = —.97), so to examine whether a predicted asymmetry in Covid-19 related knowledge was associated with actual symptom knowledge, we calculated Spearman’s rank correlation between these two variables.

### Covid-19 behavior

*Risk assessment*. ***Covid self-peer risk assessment*.** Participants were asked to estimate their risk of becoming infected with Covid-19, their risk of transmitting Covid-19, and their risk of dying if they were to become infected with Covid-19. They were then asked to make the same assessments for their average peer. All risk estimates were between 0–100%. To examine how perceived self-focused (infection risk) vs. social-focused (transmission risk) COVID-risk assessment differed across the self and other, we ran an exploratory repeated-measures 2 (target: self, other) x 2 (action-type: infection, transmission) ANOVA using the “rstatix” package [43] in R.

*Behavioral restriction*. ***Self- and peer reported behavioral restriction*.** Participants were asked to indicate how much they had limited their social interactions to reduce their likelihood of becoming infected (self-focused) and how much they had limited their social interactions to reduce their likelihood of exposing others to Covid-19 (pro-social-focused). They were also asked to indicate to what extent they felt that their average peer had done the same. All questions were answered on a scale from 0 = “Not at all” to 6 = “Extremely”.

### Specific behavior change

To assess specific behavior change, we asked participants to indicate the number of behaviors that were common pre-pandemic (e.g., going to restaurants, attending events with audiences, using ride share services) that they had limited within the past two weeks from a list of 21 behaviors (see S1 File for a full list of behaviors). We created a count variable for the number of behaviors that the participants had reported limiting, such that higher numbers indicate limiting more behaviors.

### Covid-specific worry, perceived stress, & anxiety scales

*Covid-related worry*. Participants were asked to indicate how often they worried about Covid-19 during the past two weeks via the following questions (all on a scale from 0 = “Not at all” to 6 = “Extremely frequently”): “I worried about my health”, “I worried about Covid-19”, “I worried about all the things I could do about Covid-19”, “News about all health-related topics made me worry about Covid-19”, “Changing my routine made me worry about Covid-19”, “I talked with my friends about Covid-19”, and “I felt like I had control over whether or not I would become infected with Covid-19 (reverse-scored).” These questions had good internal consistency, *a* = .81, and were combined to create a composite variable of Covid-19 related worry.

*Perceived stress*. Subjective reports of perceived stress were assessed using the validated Perceived Stress Scale (PSS [26]) from prior research. Participants indicated how often they had experienced stress over the past two weeks, with higher numbers on the scale reflecting more perceived stress. In this article, we will refer to this measure of subjective stress levels simply as “perceived stress”.

*Anxiety*. Symptoms of anxiety were assessed using the anxiety subscale of the DSM-5 Cross-Cutting Symptom Measure (DSM-5-CC [29]). Higher numbers reflect more severe anxiety over the past two-week period.

## Analysis & results

### Self-other asymmetries in Covid-19 knowledge & restriction behavior

#### Individuals are overconfident about their Covid-19 knowledge and restriction behavior relative to peers

We first hypothesized that individuals would report knowing more (H1A) and taking more preventative action (H1B) than their average peer about Covid-19, but that this would not necessarily track with reality. An exploratory one-sample t-test on perceived Covid-19 knowledge relative to one’s peers (i.e., perceived Covid-19 knowledge asymmetry) found that the mean (*M* = 3.48, *SD* = 1.15) was significantly different from the midpoint (3), *t*(1022) = 13.48, *p* < .001, *d* = .42, indicating that our sample believed that they knew more than their peers about Covid-19 (H1A). An exploratory paired t-test indicated that participants were more likely to endorse correct transmission knowledge about Covid-19 (*M* = 2.56, *SD* = .59) as compared to incorrect symptom knowledge (*M* = .52, *SD* = .73), *t*(1022) = 54.31, *p* < .001, *d* = 1.7.

Due to the nature of our sample, we can again test whether individuals’ beliefs about their average peer hit the mark. Given that the process through which the reported self-peer discrepancy was calculated was largely identical to the process of mean-centering, if individuals are well-calibrated with regard to where they stand relative to their peers, the *average* predicted self-peer discrepancy should also be around 0. That is, individuals who are restricting their behaviors less than peers should report being lower than peers, and those who are restricting more should report being higher than peers, and this would even out in an average group estimate of 0. If this estimate were higher than 0, this would mean that, on average, individuals are overestimating what they are doing relative to their peers. We found that predicted self-peer difference in behavioral restriction (*M* = 1.75, *SD* = 1.54) was much larger than actual self-peer difference in behavior restriction (*M* = .002, *SD* = 1.06) via exploratory paired t-test, *t*(1021) = 44.51, *p* < .001, *d* = 1.39 (H1B). This indicates that individuals were significantly overestimating the extent to which they are restricting their behaviors relative to their peers.

#### Individuals are optimistic about their likelihood of infection relative to peers

**Fig 1:**
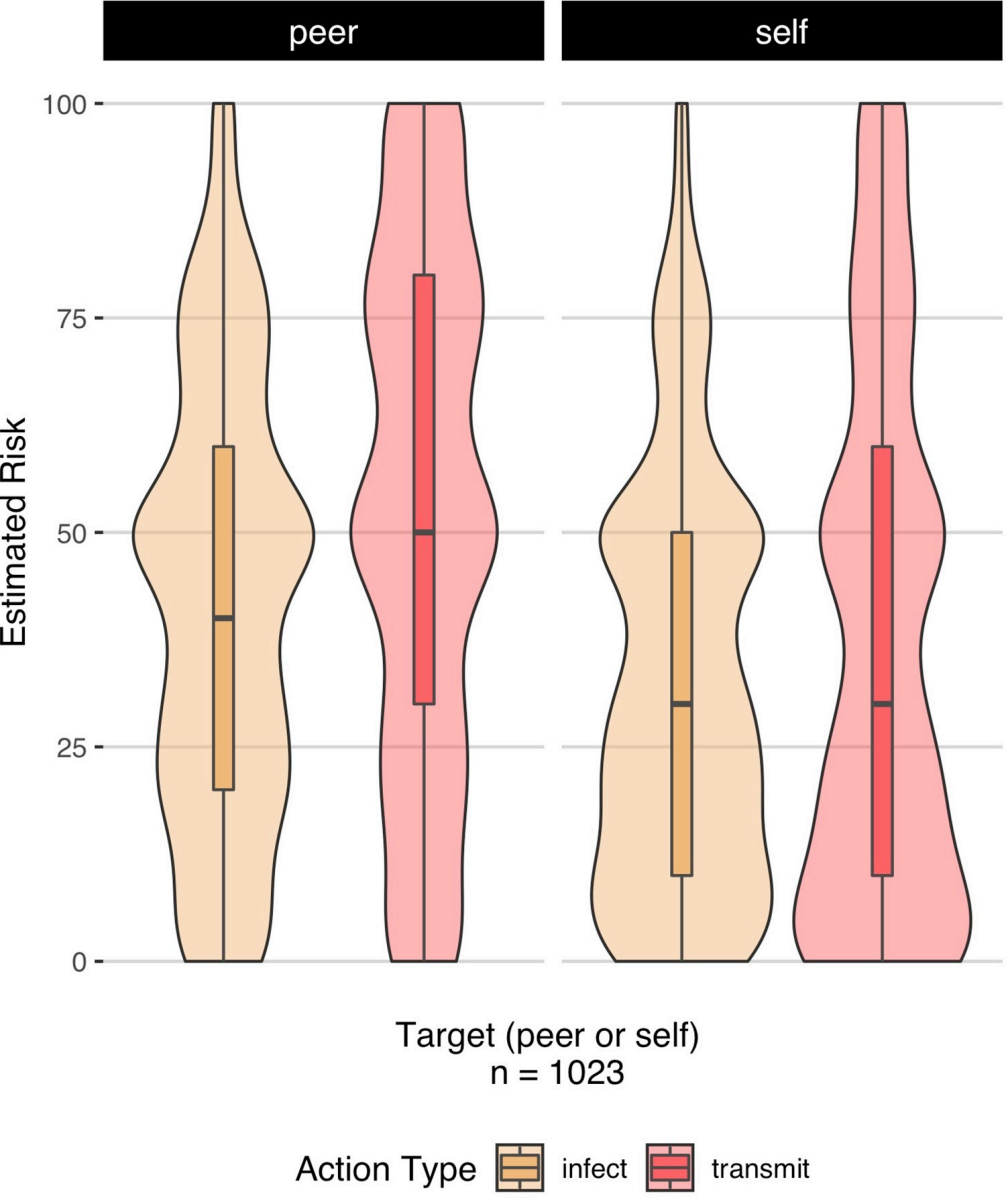
Interaction between target (self, peer) and action type (getting infected with Covid-19, transmitting Covid-19 to someone else) on estimated risk. Relative to their peers, individuals predicted that they would be less likely to get infected with Covid-19, and also less likely to transmit it to others.

We hypothesized that, consistent with other research on unrealistic optimism regarding health-related risk [44], participants would indicate that their peers were at higher risk than they were for getting infected with (i.e., self-focused) and transmitting Covid-19 (i.e., pro-social) to others (H1C). We found a significant target (2: self, peer) by action-type (2: infection, transmission) interaction (via exploratory repeated-measures ANOVA), *F*(1, 4084) = 11.35, *p* < .001, generalized eta-squared = .003 (H1C). Post-hoc, Bonferroni-corrected pairwise comparisons of this interaction indicated that in the self condition, the perceived likelihood of transmitting the virus to others was higher (*M* = 36.88, *SD* = 30.95) than perceived likelihood of getting infected (*M* = 32.63, *SD* = 24.31), *t*(1021) = 5.29, *p*_*adj*_ < .001. This was smaller than the difference observed in the peer condition, wherein the predicted likelihood of transmitting the virus to others was higher (*M* = 52.2, *SD* = 29.81) than the perceived likelihood of getting infected (*M* = 42.09, *SD* = 25.53), *t*(1021) = 15.26, *p*_*adj*_ < .001) (Fig 1). We found no differences in estimated risk of death following COVID-19 infection between self- (*M* = 11.28, *SD* = 17.53) and peer-target conditions (*M* = 11.41, *SD* = 15.95), *t*(1022) = .29, *p* = .77, *d* = 0.009 (exploratory paired t-test), suggesting that participants believed that they were as likely as peers to be negatively impacted by Covid-19, were they to contract it.

#### Relationship between self-peer asymmetries and mental health outcomes

Next, we examined the role that perceived self-peer asymmetry may play in maladaptive emotional responding. To do so, we quantified the relationships between asymmetry measures (knowledge asymmetry, behavioral restriction asymmetry) and both Covid-specific worry, perceived stress, and anxiety more generally.

*Knowledge asymmetries and mental health outcomes*. We examined the relationship between our asymmetry measures (knowledge asymmetry, behavioral restriction asymmetry) and both Covid-related worry and general anxiety (H1D). We first ran a preregistered regression model using simple linear regression (rather than multilevel as data came from the same timepoint) with Covid-related knowledge asymmetry predicting Covid-related worry. A positive relationship would indicate that individuals who believe that they know more about Covid-19 relative to their peers are also more worried about Covid-19. To examine whether knowledge asymmetry predicts Covid-19 worry specifically or perceived stress and anxiety more generally, we also ran two preregistered linear regressions with covid-related knowledge asymmetry predicting perceived stress (as assessed by the PSS) and anxiety (as assessed by the DSM-CC-5).

Turning first to Covid-specific worry, we found that, on average, our participants were moderately worried about Covid-19, *M* = 3.53, *SD* = 1.23. Consistent with our hypothesis, we found that individuals who felt that they were more knowledgeable about Covid-19 were also those that reported a higher frequency of Covid-specific worry, *b* = .21, *SE* = .03, 95% CI: [.15, .29], *t*(1021) = 6.53, *p* < .001 (H1D). To examine whether this asymmetry predicted Covid-19 worry specifically or perceived stress and anxiety more generally, we also examined the relationship between knowledge asymmetry and perceived stress (as assessed by the PSS) and reported anxiety (as assessed by the DSM-CC-5). We did not find a relationship between knowledge asymmetry and experienced perceived stress, *b* = -.07, *SE* = .19, 95% CI: [-.49, .31], *t*(1021) = .37, *p* = .71 (H1D). We did, however, find a marginally significant relationship between Covid knowledge asymmetry and anxiety, *b* = .06, *SE* = .03, 95% CI: [-.002, .12], *t*(1021) = 1.90, *p* = .057 (H1D).

*Behavioral restriction asymmetries and mental health outcomes*. To examine whether believing one is restricting behavior more than peers is related to negative mental health states (H1D), we ran a preregistered regression model (simple rather than multilevel, as data came from the same timepoint) with asymmetry in behavioral restriction predicting Covid-specific worry. To examine whether asymmetries in perceived behavioral restriction predicted Covid-specific worry specifically or anxiety more generally, we also ran two preregistered linear regressions with perceived behavioral restriction predicting perceived stress and anxiety, respectively.

We found that Covid-specific worry was significantly associated with the predicted self-peer difference in behavioral restriction, *b* = .17, *SE* = .02, 95% CI: [.12, .22], *t*(1021) = 6.90, *p* < .001 (H1D). We also found a significant relationship between perceived stress and predicted self-peer behavioral restriction, *b* = .78, *SE* = .14, 95% CI: [.49, 1.05], *t*(1021) = 5.49, *p* < .001 (H1D), and between anxiety and predicted self-peer behavioral restriction, *b* = .15, *SE* = .02, 95% CI: [.10, .19], *t*(1021) = 6.26, *p* < .001 (H1D). Taken together, this suggests that individuals who perceived greater differences in behavioral restriction between themselves and their peers are also more worried about Covid-19, and experienced greater perceived stress and anxiety.

### Self-other asymmetries in perceived Covid-19 behavior

#### Individuals believe that they are restricting their behavior more, and for more pro-social reasons, relative to peers

**Fig 2:**
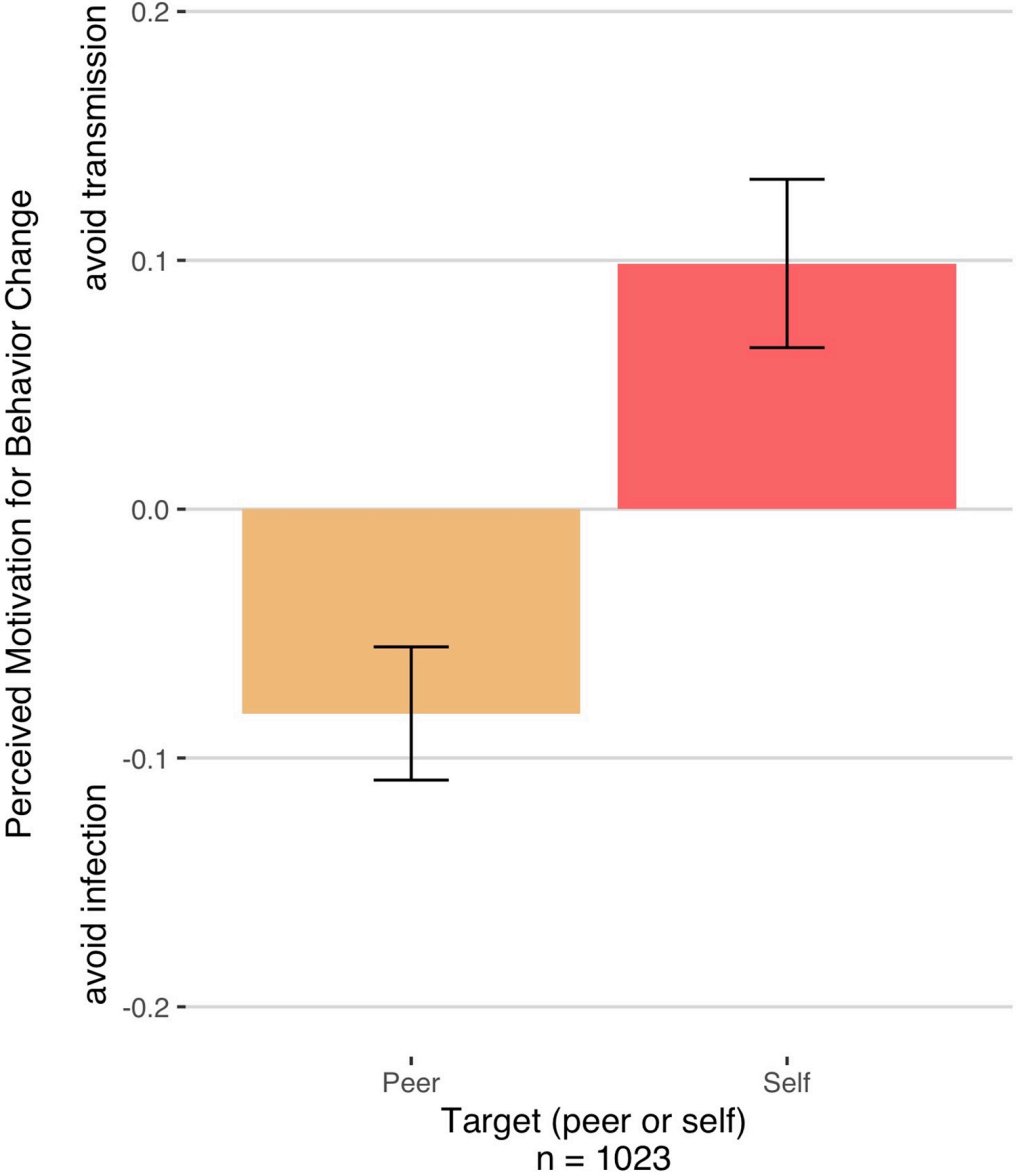
Interaction between target (self, peer) and perceived motivation for behavior change (restricting behavior to avoid infection vs. restricting behavior to avoid transmission). Individuals reported that they had limited their behaviors to avoid transmitting the others more than they had limited it to avoid getting infected. They reported that the opposite had been the case for their peers. Bars represent difference scores between transmission motivation and infection motivation.

Turning first to beliefs about behavior change, we found a significant interaction between target (self, peer) and action type (infection, transmission) on perceived behavior change (via exploratory repeated-measures ANOVA), *F*(1,4088) = 5.19, *p* = .023, generalized eta-squared = .001 (H2). Post-hoc, Bonferroni-corrected pairwise comparisons of this interaction indicated that individuals were more likely to report that they had limited their behaviors to avoid transmitting the virus to others (*M* = 5.24, *SD* = 1.21), as compared to protecting themselves from infection (*M* = 5.14, *SD* = 1.18), *t*(1022) = 2.92, *p* = .004. In contrast, they reported the *opposite* for their peers, such that they estimated that their peers were less motivated to limit their behaviors to avoid transmitting Covid-19 to others (*M* = 3.40, *SD* = 1.42), as compared to protecting themselves from infection (*M* = 3.48, *SD* = 1.25), *t*(1022) = 3.07, *p* = .002 (Fig 2). Taken together, this indicates that individuals believed that they limited their behavior in order to protect others, but that their peers had done so to protect themselves (H2).

#### Are beliefs about Covid-19 knowledge relative to peers accurate?

A Spearman’s rank correlation (rather than the pre-registered one-sample t-test due to high levels of skewness in the Covid-19 symptom knowledge variable, skewness = -0.97) showed that perceived Covid-19 knowledge asymmetry was not related to being more knowledgeable about Covid-19 symptoms: *r*_*s*_ = .04, 95% CI: [-.02, .11], *p* = .159, or general Covid-19 knowledge (via simple linear regression): *b* = .015, *se* = .03, 95% CI: [-.04, .07], *t*(1020) = .49, *p* = .625, relative to one’s peers (H3A). Taken together, this indicates that while individuals believed that they were more knowledgeable about Covid-19 than their peers, this belief did not reflect actually knowing more about Covid-19 symptoms or how the disease spreads.

**Are beliefs about behavioral restriction relative to peers accurate?** To examine whether self-other behavioral restriction assessments tracked with reality, we computed 3 difference scores, outlined in Table 1. Given that they were highly correlated (*r* = .76), we collapsed across transmission-based and infection-based restriction for the remaining analyses. For all scores, higher numbers reflect a bias towards the self-restricting more than peers (Table 1).

**Table 1:** Difference score calculations to assess each of the measured self-peer asymmetries.

Asymmetry Type	Variables
Predicted Behavioral Restriction	(Self-reported behavioral restriction −− self-estimated peer restriction)
Actual Behavior Restriction	(Self-reported behavioral restriction − average peer restriction)
Specific Behavior Change	(Number of self-reported specific behaviors −− Number of average peer specific behaviors)

#### Individuals are inaccurate about magnitude, but right about direction of perceived self-other restriction behavior asymmetry

When testing whether the asymmetry in predicted behavioral restriction (via difference score between self and peer) was associated with an asymmetry in actual behavioral restriction (via difference score between self and peer; H3B) via Spearman’s rank correlation (used rather than the pre-registered one-sample t-test due to high levels of skewness in the actual behavioral restriction variable, skewness = -1.58) between predicted and actual behavioral restriction, we found that predicted behavioral restriction and actual behavioral restriction were significantly and positively correlated, *r*_*s*_ = .55, 95% CI: [.50, .59], *p* < .001, such that individuals who thought that they were restricting behavior more than their peers generally were (H3B). Taken together, this suggests that individuals who thought they were restricting behavior more than their peers actually were, but to a lesser extent than they believed.

### Testing links between self-other asymmetries in Covid-19 knowledge and behavior and mental health outcomes

Next, we examined whether a larger self-other asymmetry in perceived behavioral restrictions is linked to more negative mental health outcomes during the Covid-19 lockdown period. Specifically, we were interested in whether anxiety symptoms arose through the experiences of Covid-specific worry and perceived stress during the pandemic by way of the perception that one personally took more action to both reduce likelihood of personal infection and transmission of the virus (H4). We conducted a set of exploratory analyses using the “lavaan” package in R [45].

To do so, we tested two indirect effects models wherein the magnitude of self-other perceived behavioral asymmetry acted as a mediator in the relationships between 1) Covid-specific worry and general anxiety, and 2) perceived stress and general anxiety (all model variables standardized). We tested these pathways as compared to the reverse (i.e., general anxiety predicting Covid-specific worry or perceived stress) as reverse pathway testing cannot always identify feasible models [46], and our proposed pathway is grounded in theoretical basis since prior research has indicated that the lockdown period has been associated with global increases in anxiety and depression [32–35]. We posited that perceived asymmetries in behavioral restriction may be one of the (likely many) variables underlying this relationship. Individuals who are more concerned about Covid-19 may also believe that they are limiting their behaviors to a greater extent than peers. Given that Covid-19 spreads due to contact between individuals, this means that peer actions can have meaningful consequences for the health of the self and others, and that this may be associated with increased feelings of anxiety during the early lockdown period.

We acknowledge the limitations of mediational claims/analyses—namely, that the scope of our (and any) study is limited such that we cannot capture all possible indirect effects that would impact the relationships from Covid-related worry or perceived stress to general anxiety early in the pandemic [47], and thus report the following in addition to the mediation analysis: 1) the correlations across all included variables so that readers may assess the association between the variables of interest (Table 2), and 2) the results of testing another potential mediator (self-other Covid-related knowledge asymmetry) assessed within the same participants at the same time. We test this additional model/alternative mediator as it is possible that individuals who are more concerned or stressed about Covid-19 may also be those who have sought out more information about it, and that having increased information about a serious disease may be associated with heightened general anxiety.

**Table 2:** Correlations between the variables of interest.

Variable	M	SD	1	2	3	4
1. General Anxiety	1.52	1.17				
2. Self-Peer Behavioral Asymmetry	1.75	1.54	.19**			
			[.13, .25]			
3. Covid-Specific Worry	3.54	1.23	.51**	.21**		
			[.47, .56]	[.15, .27]		
4. Perceived Stress	20.6	7.06	.60**	.17**	.42**	
			[.56, .64]	[.11, .23]	[.37, .47]	
5. Self-Peer Covid-19 Knowledge	3.48	1.15	.06	.20**	.20**	-.01
			[-.00, .12]	[.14, .26]	[.14, .26]	[-.07, .05]

* indicates *p* < .05

** indicates *p* < .001.

**Fig 3:**
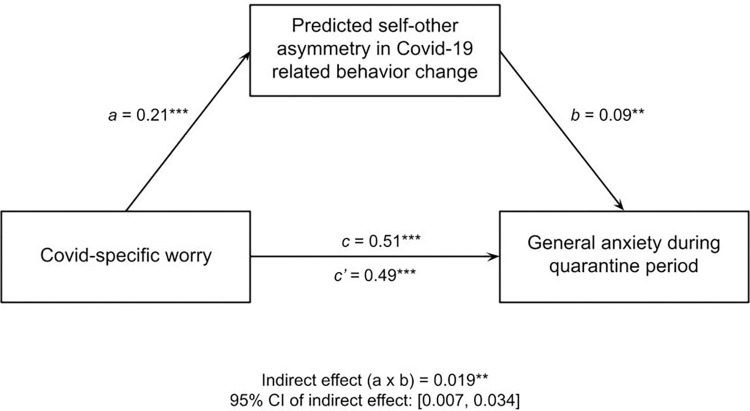
Self-other asymmetry in perceived behavioral change mediated the relationship between Covid-specific worry and general anxiety during the quarantine period.

We found that the pairwise correlations between Covid-specific worry and self-peer perceived behavioral asymmetry (*r* = .21, *p* < .001) and between self-peer behavioral asymmetry and general anxiety (*r* = .19, *p* < .001) are strong enough to partially account for the relationship between Covid-specific worry and general anxiety (*r* = .51, *p* < .001). This is consistent with a mediation model (indirect effect determined via 5000 iterations of non-parametric bootstrapping: *b* = .019, 95% CI: [.007, .034], *SE* = 0.007, *z* = 2.774, *p* = .006), but also consistent with other causal models (H4; Fig 3). This potentially indicates that while Covid-related worry was a strong predictor of general anxiety during the early lockdown period, this was due, in part, to the extent to which individuals felt that they were doing more than peers to reduce the spread of the virus.

**Fig 4:**
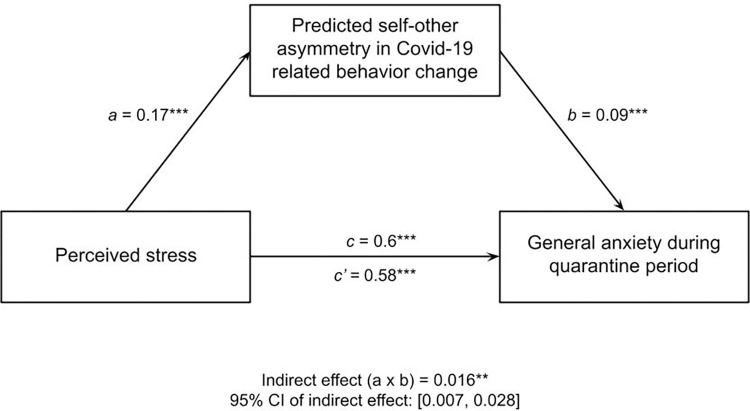
Self-other asymmetry in perceived behavioral change mediated the relationship between perceived stress (via PSS) and general anxiety during the quarantine period.

Likewise, we found that the pairwise correlations between perceived stress during the pandemic and self-peer perceived behavioral asymmetry (*r* = .17, *p* < .001) and between self-peer behavioral asymmetry and general anxiety (*r* = .19, *p* < .001) are strong enough to partially account for the relationship between perceived stress and general anxiety (*r* = .6, *p* < .001). Again, this is consistent with a mediation model (indirect effect determined via 5000 iterations of non-parametric bootstrapping: *b* = .016, 95% CI: [.007, .028], *SE* = 0.005, *z* = 2.957, *p* = .003), but also consistent with other causal models (H4; Fig 4). This suggests that, like Covid-specific worry, perceived stress was also a strong predictor of general anxiety during the early stages of the Covid-19 lockdown partially by way of estimation that one took more action to reduce spread of the virus than peers.

Unlike the relationship observed for self-peer behavioral asymmetry, we did not find that the pairwise comparison between Covid-19 related knowledge and Covid-specific worry (*r* = .20, *p* < .001) and Covid-specific knowledge and general anxiety (*r* = .06, *p* = .057) was strong enough to partially account for the relationship between Covid-specific worry and general anxiety (indirect effect determined via 5000 iterations of non-parametric bootstrapping: *b* = -.009, 95% CI: [-.022, .002], *SE* = 0.006, *z* = -1.484, *p* = .138). Likewise, we found that the pairwise comparison between Covid-19 related knowledge and perceived stress was not significant (*r* = -.01, *p* = 0.71), and that the pairwise comparison between those two variables and Covid-specific knowledge and general anxiety (*r* = .06, *p* = .057) was also not strong enough to partially account for the relationship between perceived stress and general anxiety (indirect effect determined via 5000 iterations of non-parametric bootstrapping: *b* = -.001, 95% CI: [-.006, .003], *SE* = 0.002, *z* = -0.337, *p* = .736). This suggests that while perceived self-other asymmetry in Covid-related knowledge is associated with Covid-specific worry and is significantly and/or marginally correlated with nearly all of the variables of interest, it was not an effective candidate as an alternative mediator.

## Discussion

We found evidence for substantial self-peer asymmetries regarding the Covid-19 pandemic. Individuals believe (inaccurately) that they know more about Covid-19 than their peers (H1A and H3A) and are less likely to contract and transmit the virus (H1C). Individuals also believe that they restrict their behaviors to a greater extent than their peers (H1B), although (contrary to H3B) we found that this perceived behavioral asymmetry reflected reality. Moreover, people perceived their Covid-related behaviors were more pro-socially motivated (H2) than their peers’. We found that this self-peer asymmetry, which has been associated with positive psychological outcomes in earlier research, was instead linked to negative mental health outcomes during the Covid-19 lockdown (H1D). The extent to which people thought they were restricting their behavior more than their peers (self-peer asymmetry) was strongly associated with both Covid-specific worry, perceived stress, and generalized anxiety during early stages of the pandemic (H4). Taken together, this suggests that while self-other asymmetries may be adaptive or even beneficial in typical circumstances, they may be psychologically suboptimal in challenges requiring collective action such as public health crises.

We found evidence for both accuracy and inaccuracy when estimating peer knowledge and behavior. Individuals reported that they were more knowledgeable about Covid-19 relative to their peers—a prediction not borne out in reality. Given the extent of media coverage that the Covid-19 epidemic received during the data collection period, it is likely that individuals were often exposed to information about the pandemic and indeed *were* knowledgeable about the disease. However, they failed to recognize that their peers were also likely inundated by Covid-19 related news, and were likely equally knowledgeable.

We also found that individuals were correct about the direction, but overestimated the magnitude, of the self-peer asymmetry in behavior. Individuals believed that they were restricting their behavior more than their peers to a greater extent than was actually the case. However, individuals who were restricting their behavior more (or less) than peers were accurate about their relative standing. This suggests that individuals are correctly identifying where they stand relative to peers in terms of limiting behavior, but are perhaps too generous when it comes to determining how *far* above their peers they actually are. Consistent with accounts of unrealistic optimism [45, 47], we found that individuals predicted that they were less likely than their peers to get infected with or transmit Covid-19 to others. Individuals were also more likely to claim that they were restricting their behavior for pro-social reasons than were their peers’. This is consistent with research indicating that individuals tend to amplify the extent to which they are above-average in positive and moral domains [11], and report being more morally-motivated relative to their peers [3].

While self-serving bias is often associated with maximizing happiness and self-esteem, we found that this belief pattern was associated with greater amounts of anxiety and perceived stress in the context of Covid-19. This could be due to a number of factors. Feeling a lack of situational control is associated with increased negative affect [48], and it is possible that participants who were more pessimistic about their peers felt less situational control and more uncertainty during Covid-19. This is consistent with our findings that behavior-related self-peer asymmetries, but not knowledge-related ones, were relevant in linking both perceived stress and Covid-specific worry with anxiety levels. Research has also found that while self-serving bias may facilitate coping in traumatic and negative situations [49], it is also associated with being less liked by others [49–52]. It is possible that individuals who exhibited greater self-other asymmetry may have had less social support, increasing anxiety during a stressful time. Situational control, uncertainty, and perceived social support should be examined in future research as possible causal factors.

Prior work has found that anxiety tends to be negatively associated with self-enhancing biases [40]. However, in the context of a public health threat, the relation between detrimental health outcomes and believing that one is doing more than peers makes sense when individual health outcomes are reliant on the actions of others. Although trust was not explicitly measured, our findings that anxiety levels are predicted by Covid-specific worry and perceived stress by way of perceived self-peer asymmetry in prosocial public health actions may be indicative of reduced interpersonal trust. The perception that prosocial norms have been violated by others while being upheld by the self (which we find can be accurate) may result in negative mental health outcomes. This is consistent with past work finding that lack of support reciprocity within couples tends to be associated with reduced relationship satisfaction [53], and that greater interpersonal trust is associated with reduced symptoms of depression and anxiety and enhanced physical health [54].

Furthermore, within the context of the pandemic specifically, this lack of trust was not without reason, given the politicization of public health behaviors that resulted in undercompliance with prosocial public health regulations [55]. Investigation of the role of intergroup dynamics in associations between self-other asymmetries and mental health, including (but not limited to) belief in the cooperation of political in- and out-groups, represents an important future direction. For example, people may actually be more negatively impacted by lack of perceived cooperation from in-group than out-group members, as they may have stronger baseline trust in in-group members [56]. Interestingly, we find here that people *underestimate* the cooperative behaviors of their own peers. This is perhaps because, although adaptive [57], practicing cooperation and pro-sociality can be demanding [58]; as with other effortful but personally-valued tasks, this difficulty may be recognized among others, but not the self [3]. Finding ways to demonstrate action-focused care for one another and build interpersonal trust may both benefit personal mental health and enhance the mitigation of public-level health threats.

### Limitations

Despite its strengths, this study is not without shortcomings. One limitation is the ambiguity about whom participants were thinking of when asked about an “average peer”. Although this framing is fairly consistent with past research on self-other asymmetries (e.g., prediction of personal behavior and that of an anonymous peer or broadly-construed “peers”, [3]), some prior work has found that self-other asymmetries are mitigated as targets become more individuated [59]. Thus, results may not be as pronounced if individuals compared themselves to an individuated peer. Although they were members of the same university community which gives way to formation of many relationships and shared experiences (e.g., exposure to the same university Covid-19 communications and regulations), we cannot be completely certain that individuals were thinking of their university-specific peers when making self-peer assessments. Although university peers would most likely be a salient peer community, it is possible that participants were perhaps comparing themselves to their hometown friends, Instagram followers, or the neighbors down the street. It is worth noting that this would only change the results with regard to self-peer accuracy, and not the general conclusions with regard to mental health outcomes. Similarly, it is possible that order effects may have influenced our results, as participants all responded to scale measures presented in the same order.

It is also unclear what information motivated the self-enhancing assessments observed here. Many factors have been identified as underlying self-serving bias, including failure to integrate base-rate information when evaluating the self [3], a failure to receive or integrate objective feedback [60], having insufficient skills or expertise to be able to recognize one’s own incompetence [61], and motivated autobiographical recall [62]. It is possible that when assessing peer behaviors, individuals tended to rely more on statistical information about things like mask compliance, and instead, when assessing their own behaviors, selectively recalled times that they socially distanced or social invitations that they declined. It is also likely that rational factors give rise to this bias—individuals have more information about the extent to which they have limited or changed behavior following the Covid-19 epidemic as compared to the information they have about their peers.

As described in detail in the Introduction, some prior work [17] has come from data collected in a later wave of the same longitudinal project as the present article. The present article investigates unique research questions and analyzes distinct data (collected at separate timepoints) relative to the prior work [17]. The articles are thus independent both in terms of their research foci and data examined.

It is also worth noting the limitations of mediation analysis in terms of identifying causal mechanisms–as mentioned in our results, there are many potential mediators in the relationship between Covid-19 specific worry and anxiety (e.g., financial stress, job loss, health concerns), and identifying perceived self-other asymmetry as a potential mediator between Covid-19 specific worry and general anxiety does not preclude the existence or identification of other potential mediators. Finally, it is worth noting that our results come from participants within a university located in the United States and are thus limited in their generalizability in that regard. This is particularly true given known differences about Covid-19 responses and attitudes in the U.S. and other nations (e.g., trust in others, [22]).

## Conclusion

Adding to a growing body of work on the implications of the Covid-19 pandemic for mental health, including rising levels of in loneliness [63], substance use [64], and depression and anxiety rates [65], we identify self-serving biases in the space of public health behavior as one possible mechanism linking perceived stress and pandemic-specific worry with anxiety levels. Future research should examine the long-term effects of self-serving bias related anxiety—does it function as a motivator for pro-social action, or does it increase the likelihood of burnout? Given that humans will face no shortage of collective action problems in the foreseeable future, understanding how the psychological processes that we study in the lab promote, prevent, and preclude collective action has perhaps never been so important.

Future research should further explore the impacts of interpersonal trust (or lack thereof, as suggested through self-serving bias here) on more positive facets of mental health, including resilience and flourishing. Furthermore, future research should pinpoint the extent to which intergroup contexts influence mental health outcomes related to trust in others’ prosociality [56]. Lastly, interventions and publicly-accessible education regarding the existence and influence of self-serving biases on mental health may help to reduce their negative impacts. In clinical settings, providing this information may facilitate cognitive restructuring processes [66].

Finding ways to promote action-oriented forms of cooperation and community care may be crucial preserving individuals’ mental health in the face of collective challenges. Continued focus on interventions to understand self-serving biases, develop interpersonal trust, and build community may not only support progress, but also enhance psychological health.

### Open practices

Hypotheses and their corresponding analyses—unless indicated otherwise (i.e., labeled exploratory)—were preregistered (https://aspredicted.org/blind.php?x=/HGQ_8XB). All data and analysis scripts/output are available via Open Science Framework (OSF): https://osf.io/hpv43/?view_only=fec42fadb67240aa8d3c8780d002bc3f.

## Supporting information

S1 File Covid-restricted actions. (DOCX)
