# Supplementary material for: When thinking you are better leads to feeling worse: Self-other asymmetries in pro-social behavior and increased anxiety during Covid-19
Source: PLoS One. 2023 Sep 14;18(9):e0291329. doi: 10.1371/journal.pone.0291329 (PMC10501629; doi:10.1371/journal.pone.0291329)
Supplement: S1 File — (DOCX) [file pone.0291329.s001.docx]

**Supporting Information**

To examine whether beliefs about the extent to which individuals restricted their behavior relative to their peers related to actual differences in behavior change, we examined self-peer differences in the number of activities that participants had reported limiting over the past two weeks. We found that on average, participants reported limiting 14.75 (*SD*  = 3.58) behaviors. We found that predicted behavioral restriction relative to one’s peers was positively correlated with the number of behaviors one reported limiting relative to peer group averages, *r* = .28, *t*(1020) = 9.30, *p*  < .001.

**S1. Table. Covid-restricted actions.**

**
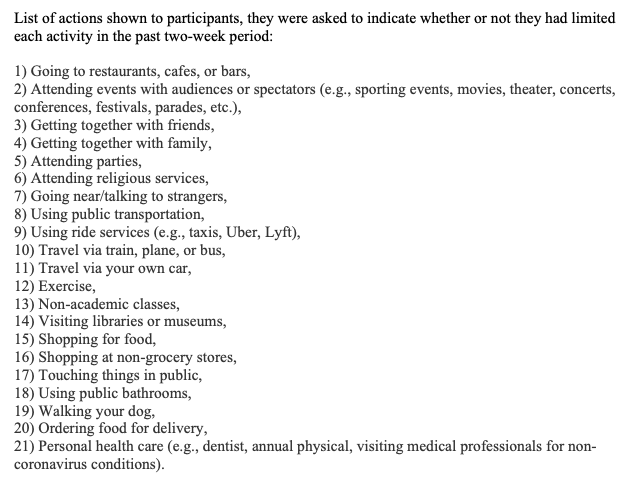
**
